# Network meta-analysis of the effects of traditional Chinese fitness exercises on mental health and physical benefits among college students

**DOI:** 10.3389/fpsyt.2026.1921373

**Published:** 2026-10-09

**Authors:** Nan Wang, Hao Hong, Maolin Dong, Yaoshu Li, Lisha Lan, Junjian Zheng

**Affiliations:** 1 Wushu College, Henan University, Kaifeng, China; 2 Department of Basic Teaching, Shandong Vocational Animal Science and Veterinary College, Weifang, China; 3 Department of Police Physical Education, The National Police University for Criminal Justice, Baoding, China

**Keywords:** body composition, cardiorespiratory function, college students, mental health, network meta-analysis, traditional Chinese fitness exercises

## Abstract

**Background:**

Traditional Chinese fitness exercises may benefit college students’ mental and physical health, but their comparative effectiveness across exercise modalities and outcomes remains unclear.

**Objective:**

To compare traditional Chinese fitness exercises across mental health, body composition, and cardiorespiratory outcomes in college students.

**Methods:**

Eight databases were searched from inception through September 8, 2026. Eligible RCTs in college students were assessed with RoB 2 and synthesized using Bayesian network meta-analysis.

**Results:**

A total of 69 RCTs involving 6,065 college students were included. For depression, Tai Chi (TC, 95.40%), Yijin Jing (YJJ, 85.40%), and Baduanjin (BDJ, 69.23%) ranked highest, with several significant pairwise comparisons. For anxiety, TC significantly outperformed blank control (BC) and ranked highest (88.82%). Other traditional exercises (OTE, 84.53%), Wuqinxi (WQX, 67.72%), and Liuzijue + physical intervention (LZJ + PI, 65.82%) ranked highest for vital capacity (VC); Xingyiquan (XYQ, 82.89%) and Mawangdui Daoyinshu (MWDY, 82.07%) for VO_2_max; and OTE (82.52%) and XYQ (78.81%) for the step test index. TC ranked highest for body fat rate (BFR, 86.35%), whereas LZJ (81.27%), WQX (72.38%), and PI (65.44%) ranked highest for body mass index (BMI). Surface under the cumulative ranking curve (SUCRA) rankings should be interpreted alongside effect sizes and 95% CrIs.

**Conclusion:**

Traditional Chinese fitness exercises may provide multidimensional benefits, with comparative advantages varying by outcome. Exercise selection should be outcome-oriented rather than based on a single universally optimal modality.

**Systematic review registration:**

https://www.crd.york.ac.uk/PROSPERO/view, identifier CRD420261290192.

## 1 Introduction

College students are a high-risk population concurrently exposed to depression, anxiety, and declining physical fitness. From the perspective of mental health, the global pooled prevalence rates of depression and anxiety among college students are 33.6% and 39.0%, respectively (1). Thus, psychological problems have become an important public health issue in this population. Meanwhile, physical fitness problems are equally concerning. Studies have shown that first-year university students gain an average of approximately 1.36 kg during their first year, and over a longer time frame, the overall weight gain during the college years is about 1.55 kg, with an increase in body fat rate (BFR) of about 1.17% (2, 3). The overall levels of physical activity and physical fitness among college students are not ideal (4). Among Chinese college students, physical fitness level is substantially correlated with mental health. A higher fitness level is generally associated with lower scores on psychological problems (5). These lines of evidence suggest that psychological distress and physical decline in college students are not independent of each other. Rather, they tend to co-occur and interact. Therefore, comprehensive intervention evaluations targeting depression, anxiety, and indicators like body mass index (BMI), BFR, and cardiorespiratory function have clear public health significance and practical value.

Exercise is a core non-pharmacological strategy for improving mental health and promoting physical benefits. However, the existing literature on exercise interventions for college students is characterized by substantial methodological and clinical heterogeneity (6, 7). Recent systematic reviews have reported considerable variations in exercise modality, intervention duration, session frequency and duration, exercise intensity, and the psychological outcomes used for assessment (6–8). Moreover, emerging dose–response evidence suggests that exercise intensity and accumulated exercise dose may modify the magnitude of mental health benefits (9). Such heterogeneity makes it difficult to directly translate pooled evidence into standardized and reproducible exercise prescriptions for university settings (6, 10–12). Compared with general aerobic or resistance training, traditional Chinese fitness exercises integrate body regulation, breath control, and mind focus, making them more suitable for low-cost, scalable, and mind-body integrated interventions in university settings. Research has demonstrated that Baduanjin (BDJ) can improve physical fitness, BMI, lung function, as well as alleviating depression and anxiety among college students (10–12). Tai Chi (TC) can also alleviate anxiety and depression in this population (13). Yijin Jing (YJJ) deserves particular attention. Although high-quality randomized evidence in college student samples remains relatively limited, existing studies have shown that YJJ can improve emotion-related outcomes and enhance lung function, physical activity, and health-related quality of life (14). From a mechanism perspective, YJJ integrates repeated muscle–tendon stretching and coordinated whole-body movements with regulated breathing and sustained attentional focus. The musculoskeletal component may improve physical function through repeated stretching and dynamic loading. Previous evidence has shown beneficial effects on muscle strength and physical performance (15). In addition, the coordination of movement and breathing may contribute to improved pulmonary function and exercise tolerance. Evidence from randomized trials has shown improvements in pulmonary function, physical activity, and health-related quality of life following YJJ practice (14). At the psychological and neural levels, the combination of controlled breathing, meditative attention, and movement regulation may facilitate emotion regulation and cognitive control. This interpretation is supported by evidence of improved emotion-regulation self-efficacy after YJJ training (14) and by an electroencephalogram (EEG)-based randomized trial reporting changes in brain functional network characteristics accompanied by reductions in depressive symptoms (14). These interacting physical, respiratory, and psychological pathways may provide a plausible basis for the simultaneous mental and physical health benefits of YJJ, although mechanism evidence specifically derived from healthy college students remains limited. In addition to YJJ, other exercises like Liuzijue (LZJ), Wuqinxi (WQX), and Mawangdui Daoyinshu (MWDY) have also demonstrated positive effects on lung function, exercise capacity, body composition, or metabolic risk factors in other populations (16–18). Although existing evidence suggests that traditional Chinese fitness exercises are “possibly effective overall”, it is still insufficient to answer the more critical questions in university practice: which exercise is superior among different exercises, and on which types of outcomes are their advantages manifested?

Although accumulating evidence suggests that traditional Chinese fitness exercises may benefit both the mental and physical health of college students, the existing evidence base remains fragmented (19). Previous systematic reviews have either evaluated qigong or traditional Chinese mind–body exercises as broad intervention categories (19), focused on a single exercise modality such as TC (20) or BDJ (21), or primarily examined psychological outcomes such as depression and anxiety (22, 23). Recent evidence has also synthesized physical-health outcomes of traditional Chinese mind–body training in university students (24). However, mental and physical health outcomes have generally been evaluated separately, and evidence simultaneously comparing multiple specific traditional Chinese exercises across both domains remains limited (23, 24). Consequently, it remains unclear whether different traditional Chinese fitness exercises exhibit outcome-specific advantages across psychological and physical domains. Therefore, the present study used network meta-analysis to integrate direct and indirect evidence and systematically compare the relative effects of different exercises on depression, anxiety, BMI, BFR, vital capacity (VC), maximal oxygen uptake (VO_2_max), and the step test index in college students, thereby providing a more differentiated evidence base for exercise selection and prescription development in university populations.

## 2 Methods

This study was designed, conducted, and reported in strict adherence to the Preferred Reporting Items for Systematic Reviews and Meta-Analyses (PRISMA) statement and its extension for network meta-analyses (**Supplementary File S1**) (25). The protocol was prospectively registered on the international prospective register of systematic reviews (PROSPERO) under the registration number CRD420261290192.

### 2.1 Search strategy

PubMed, Embase, Cochrane Library, Web of Science, CNKI, Wanfang, VIP, and CBM were searched from their inception to January 4, 2026. During the revision process, the original search strategies were rerun to identify newly published or newly indexed studies, and the search was updated through September 8, 2026. The search was restricted to Chinese and English literature. The search strategy combined medical subject headings (MeSH) and free-text terms, and the search strings were appropriately adjusted according to the search rules of different databases. The search content mainly comprised three parts: study population (college students), intervention (traditional Chinese fitness exercises), and study design (randomized controlled trials (RCTs)). The Chinese search terms mainly encompassed “college student”, “Tai Chi”, “Baduanjin”, “Yi Jin Jing”, “Wu Qin Xi”, “Liuzijue”, “qigong”, “Daoyin Yangsheng Gong”, “traditional Chinese fitness exercise”, and “random”. The English search terms mainly included “undergraduate”, “student*”, “Ba Duan Jin”, “Baduanjin”, “Dao Yin”, “Five Animal Frolics”, “Liu Zi Jue”, “Liuzijue”, “qigong”, “Tai Chi”, “traditional Chinese exercise”, “Wu Qin Xi”, “Yi Jin Jing”, “random*”, and “RCT”. In addition, the reference lists of included articles and relevant reviews were traced to minimize potential omissions. The search strategy is detailed in **Supplementary File S2**.

### 2.2 Inclusion and exclusion criteria

The inclusion and exclusion criteria were established according to the population, intervention, comparison, outcome, study design (PICOS) principle. The included studies were required to meet the conditions below: (1) study population: general university students, including undergraduates, postgraduates, and university freshmen; (2) intervention: the intervention group received traditional Chinese fitness exercises, including but not limited to TC, BDJ, YJJ, WQX, LZJ, and Daoyin Yangsheng Gong (DYYS). The control group received blank control (BC), psychological intervention (PI), or regular exercise (RE); (3) study type: RCTs; (4) at least one of the following types of outcome measures was reported: mental health indicators (anxiety, depression), body composition indicators (BMI, BFR), or cardiopulmonary function indicators (VC, VO_2_max, step test index).

The exclusion criteria were outlined below: (1) duplicate publications (the version with the most complete information was retained); (2) non-RCTs, such as reviews, meta-analyses, conference abstracts, case reports, animal studies, and study protocols; (3) studies that did not report the pre-specified outcome measures of this study, or that had incomplete data from which effect sizes could not be extracted; (4) studies not published in Chinese or English.

### 2.3 Literature screening and data extraction

The retrieved records were imported into EndNote. Two researchers (N.W. and H.H.) independently performed literature screening according to the pre-established inclusion and exclusion criteria. First, duplicate records were removed. Then, the titles and abstracts were examined to eliminate ineligible studies, and the full texts were further reviewed to determine the final included studies. Any disagreements between the two researchers were resolved through discussion and, when necessary, adjudicated by a third researcher (M.D.).

Data were independently extracted by two researchers (N.W. and H.H.). The acquired information mainly encompassed the first author, year of publication, country or region, baseline characteristics like sample size, sex, age or grade level of the study participants, type, duration, and frequency of intervention, duration per session, control measures, and relevant outcome data. The acquired data were entered into Microsoft Excel for organization and verification. For studies with incomplete data, information was extracted from the main text, tables, and **Supplementary Materials**. When necessary, the original authors were contacted to obtain relevant data. If the same study had multiple publications, only the version with the most complete information was included.

### 2.4 Quality assessment

The risk of bias of the included RCTs was independently evaluated by two researchers (M.D. and Y.L.) utilizing the Cochrane Risk of Bias Tool version 2 (RoB 2) (26). The evaluation covered five domains: the randomization process, deviations from intended interventions, missing outcome data, measurement of the outcome, and selection of the reported result. Each domain was identified as having “low risk”, “some concerns”, or “high risk”. Any divergences between the two researchers were addressed through discussion and adjudication by a third researcher (N.W.). The final evaluation results were presented as a risk of bias graph.

### 2.5 Statistical analysis

Statistical analyses were implemented utilizing R and Stata. For continuous variables, depending on whether the measurement units of the outcome indicators were consistent, the mean difference (MD) or standardized mean difference (SMD) was used as the effect measure, and the corresponding 95% credible interval (CrI) was reported (27). A network meta-analysis was carried out within a Bayesian framework to synthesize direct and indirect comparative evidence. First, a network plot was created utilizing Stata to illustrate the evidence structure among different interventions (28). Subsequently, model fitting was performed using R, with 500,000 iterations, of which the first 20,000 were used as burn-in. In addition, a Gelman-Rubin diagnostic value < 1.1 was set as the criterion for chain convergence. To ensure the robustness of the results, we appraised the convergence of the network meta-analysis by examining the stability of trace and density plots. Between-study heterogeneity was primarily assessed using the posterior between-study standard deviation (τ) and variance (τ²), together with their corresponding 95% CrIs. Global inconsistency was assessed by comparing the deviance information criterion (DIC) and residual deviance between the consistency and inconsistency models (27, 29). The I² values generated using the GeMTC ANOHE procedure were used only as supplementary descriptive measures of heterogeneity and were not used to assess network inconsistency. Pairwise comparisons among different exercises were presented using a league table. Intervention ranking was evaluated leveraging the surface under the cumulative ranking curve (SUCRA). A larger SUCRA value indicated a higher ranking. However, this should not be equated with real-world clinical benefit. Therefore, for interpreting the clinical significance, absolute effect data were also considered, and the magnitude of difference, precision of effect, and acceptability were comprehensively weighed to avoid the misinterpretation of “high ranking but minimal benefit” (30). In addition, comparison-adjusted funnel plots were generated to assess potential small-study effects or publication bias (28).

## 3 Results

### 3.1 Literature search and screening process

The initial search yielded 4,297 records. After the elimination of 895 duplicate records, 3,402 records remained for title and abstract screening. In the initial screening phase, 3,281 records were excluded, mainly due to inappropriate study design, ineligible study population, inappropriate intervention, animal studies, reviews, or meta-analyses. Totally 121 records met the criteria after initial screening. Among these, full texts of 10 records could not be obtained, 30 records did not match the study purpose, and 12 records had no relevant data. Finally, 69 studies were included. The literature screening process is illustrated in **Figure 1**.

**Figure 1:**
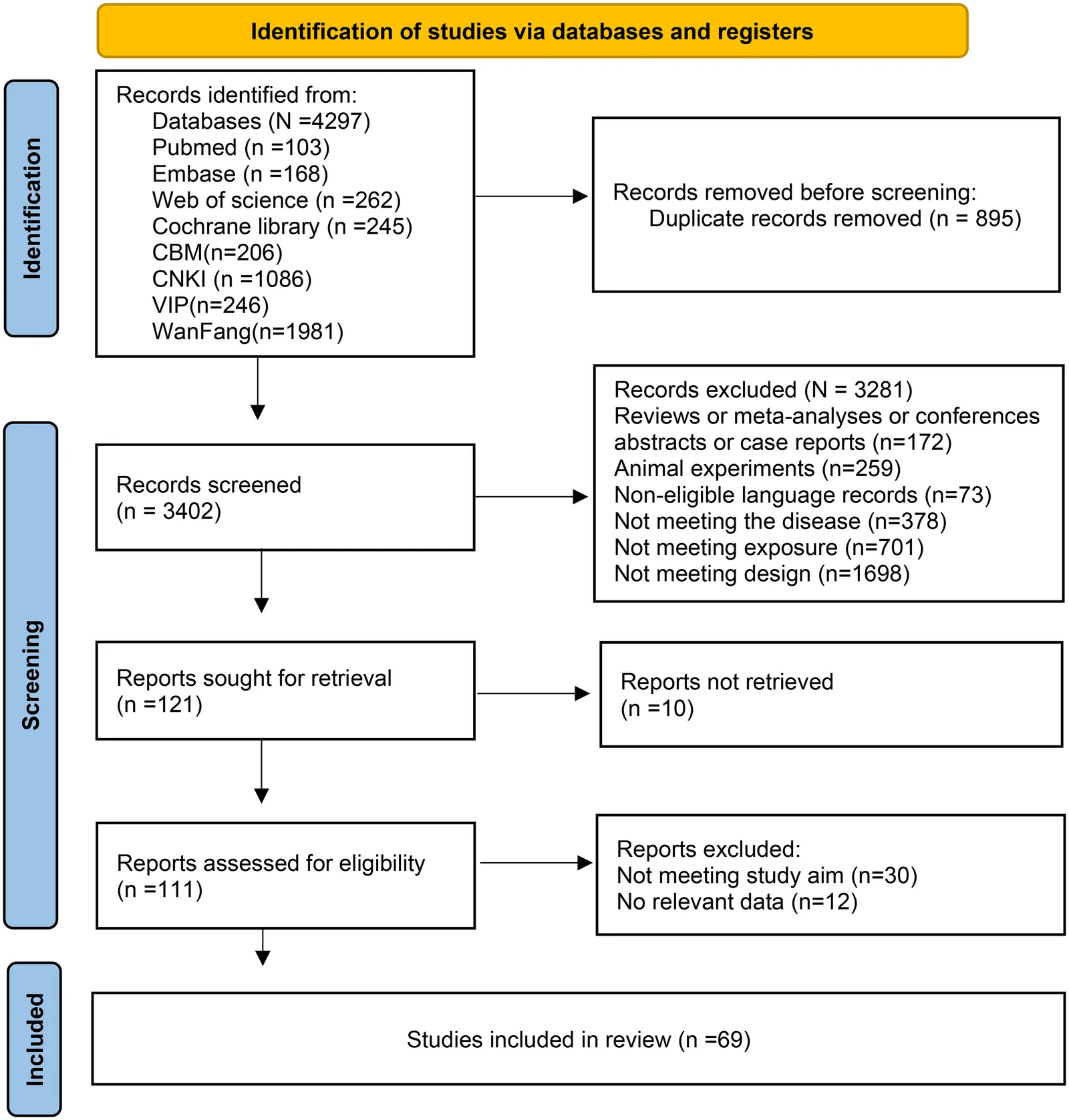
Literature screening flowchart.

### 3.2 Basic characteristics of the included studies

In total, 69 (10–13, 31–95) studies were finally incorporated, involving 6,065 individuals, published from 2007 to 2026. All studies were conducted in China, covering multiple provinces and cities including Beijing, Shanghai, Guangdong, Shandong, Anhui, Jiangsu, Shanxi, Hunan, Henan, Liaoning, Hubei, Zhejiang, Guangxi, Guizhou, Sichuan, Jiangxi, Chongqing, Hainan, Hebei, and Tianjin. All participants were university students, mainly aged between 18 and 24 years. The interventions encompassed TC, BDJ, YJJ, WQX, LZJ, DYYS, and other traditional exercises (OTE). The control measures were primarily BC, health education or PI, and exercise intervention. The intervention duration ranged from 4 to 40 weeks, with 8 to 16 weeks being the most common. The basic characteristics of the included studies are detailed in **Table 1**.

**Table 1:** Baseline characteristics.

Author/year	Country	Sample size		Age		Proportion of females		Intervention		Duration	Outcome measures
		T	C	T	C	T	C	T	C		
Chen Y/2024 (31)	China/Guizhou	50	50	18-22	18-22	50.0	50.0	MWDY	BC	12	Vomax, maxBMI
Cao J/2018 (32)	China/Hubei	20	20	/	/	/	/	TC	RE	12	BMI, BFR
Cao YS/2012 (33)	China/Hebei	80	80	19.41 ± 0.95	19.25 ± 7.77	37.5	37.5	OTE	RE	12	BMI, Step test index, VC
Bi YY/2020 (34)	China/Anhui	30	30	/	/	66.7	63.3	WQX	PI	10	BMI, Step test index, VC
Zeng SJ/2019 (35)	China/Guangdong	24	23	20.46 ± 2.04	20.65 ± 1.99	37.5	54.2	BDJ	BC	12	BMI, BFR
Cen JX/2019 (36)	China/Beijing	18	18	/	/	100.0	100.0	TC	BC	16	Depression
Chen X/2022 (37)	China/Shanghai	18	19	22.44 ± 3.20	21.98 ± 3.31	50.0	52.6	BDJ	BC	8	Anxiety, Depression
Chen YR/2018 (38)	China/Hainan	30	30	20.13 ± 1.11	20.13 ± 1.11	50.0	50.0	TC	BC	12	BMI, VC
Cheng X/2016 (39)	China/Chongqing	14	14	21.2 ± 1.2	20.9 ± 1.6	50.0	50.0	WQX	BC	12	BDI-Y, Depression
Dou YT/2019 (40)	China/Beijing	20	20	20.10 ± 2.27	20.06 ± 2.01	100.0	100.0	TC	BC	16	BMI, BFR, Step test index, VC
Du XX/2016 (41)	China/Shanxi	20	20	20.08 ± 2.16	19.21 ± 2.04	100.0	100.0	TC	BC	12	BMI, BFR, Step test index, VC
Hang B/2008 (42)	China/Beijing	32	32	18.6	18.4	100.0	100.0	TC	RE	14	BDI-Y
He SC/2021 (43)	China/Guangdong	34	C1 = 33/C2 = 33	19.40 ± 1.50	C1 = 19.47 ± 1.22/C2 = 19.70 ± 1.21	/	/	TC	C1=RE/C2BC	12	BMI, VOmax, BFR
Jia SY2008 (44)	China/Zhejiang	12	12	20.38 ± 0.72	20.92 ± 1.61	/	/	TC	OTE	12	VOmax
Jiao XX/2021 (45)	China/Anhui	40	40	20.0 ± 1.3	20.0 ± 1.3	/	/	WQX	RE	16	Depression, Anxiety, BMI, VC, Step test index
Jing JH/2013 (46)	China/Shanxi	40	C1 = 40/C2 = 40	19-25	C1 = 19-25/C2 = 19-25	/	/	LZJ	C1=BDJ/C2=BC	24	BMI, Step test index
Ke XJ/2019 (47)	China/Zhejiang	20	17	19.4 ± 0.5	19.5 ± 0.6	100.0	100.0	BDJ	BC	10	Anxiety, Depression
Li CX/2014 (48)	China/Hunan	20	20	/	/	60.0	60.0	BDJ	BC	40	Depression
Li K/2023 (49)	China/Chongqing	24	24	18-26	18-26	100.0	100.0	WQX	PI	12	Depression-24, STDS-Y, Depression, Anxiety
Li Q/2024 (50)	China/Hubei	25	25	17.68 ± 0.69	17.72 ± 0.74	76.0	68.0	YJJ	BC	8	Depression, BDI-Y
Liu C/2016 (51)	China/Beijing	30	28	21	20	53.3	35.7	OTE	BC	4	BMI, VC
Liu YJ/2013 (52)	China/Sichuan	36	36	/	/	/	/	YJJ	TC	8	Depression, SDS
Lu MF/2023 (53)	China/Jiangsu	15	15	20.67 ± 0.90	21.00 ± 0.97	100.0	100.0	TC	BC	12	BMI
Mao HN/2008 (54)	China/Shanxi	52	52	18—20	18—20	100.0	100.0	TC	BC	18	Anxiety, Depression
Meng B/2023 (55)	China/Beijing	20	20	/	/	/	/	BDJ	BC	12	Depression, Depression, Anxiety
Min Y/202 (56)4	China/Harbin	35	35	20.47 ± 0.99	20.27 ± 1.25	/	/	YJJ	RE	6	BMI, VC
Niu W/2011 (57)	China/Liaoning	64	65	/	/	48.4	50.8	BDJ	BC	12	BMI, Step test index, VC
Pei Y/2018 (58)	China/Jiangsu	25	24	18-25	18-25	100.0	100.0	BDJ	YJJ	12	Anxiety, Depression, VC
Qin Y/2011 (59)	China/Shanghai	54	54	18-21	18-21	75.9	72.2	OTE	/	12	BMI
Wang QY/2017 (60)	China/Guangxi	15	15	/	/	0.0	0.0	TC	OTE	12	VO2max/kg
Wang L/2021 (61)	China/Anhui	30	30	/	/	100.0	100.0	TC	RE	12	BMI, BFR, Step test index, VC
Wei QB/2017 (62)	China/Jiangsu	60	60	/	/	/	/	YJJ	BC	12	Depression, Anxiety, VC, Step test index
Wang M/2020p63[	China/Anhui	30	30	/	/	/	/	WQX	BC	24	Depression
WEI JJ/2007 (64)	China/Beijing	13	12	21.62 ± 1.45	21.58 ± 1.44	0.0	0.0	TC	RE	8	BMI, VO2max, BFR, VC
Yan J/2019 (65)	China/Beijing	36	36	18-25	18-25	/	/	OTE	BC	12	Anxiety
Yang YQ/2021 (66)	China/Shandong	20	C1 = 20/C2 = 20	/	/	/	/	TC	C1=RE/C2=BC	8	Depression
Yao L/2014 (67)	China/Shanghai	15	15	/	/	/	/	LZJ+PI	PI	24	Depression, VC
Yu ZS/2017 (68)	China/Jiangxi	23	23	18-23	18-23	100.0	100.0	BDJ	BC	16	BMI
Zhang DW/2023 (69)	China/Tianjing	34	34	19.46 ± 0.68	19.54 ± 0.73	0.0	0.0	BDJ	BC	8	BMI, BFR
Zhang G/2019 (70)	China/Shanxi	46	42	/	/	/	/	BDJ	BC	9	BFR, VC
Zhang YZ/2021 (71)	China/Shanxi	30	30	/	/	/	/	BDJ	BC	8	SAS, SDS
Zhang Y/2025 (72)	China/Tianjing	20	20	18-22	18-22	/	/	BDJ	RE	8	BMI, BFR
Jiang J/2024 (73)	China/Hunan	40	40	/	/	/	/	WQX	BC	12	Depression, Anxiety
Li W/2015 (74)	China/Shandong	180	180	18.75 ± 1.05	18.75 ± 1.06	50.0	50.0	WQX	RE	18	BMI, VC, Depression, Anxiety
Tan ZG/2020 (75)	China/Hunan	35	35	/	/	/	/	BDJ	BC	18	Depression, VC
Wang YN/2022 (76)	China/Guangxi	40	40					BDJ	RE	12	Depression, Anxiety, BMI, VC
Wei L/2024 (77)	China/Guangdong	76	76	19-21	19-21	总38.16	总38.16	BDJ	BC	18	Depression
Guo TR/2021 (78)	China/Liaoning	30	30	/	/	/	/	BDJ	BC	12	Depression, Anxiety
Jiang Y/2020 (79)	China/Anhui	45	45	19.31 ± 1.07	19.25 ± 1.13	62.2	62.2	WQX	PI	10	VC
Li JQ/2025 (80)	China/Tianjing	15	C1 = 15/C2 = 15	22.73 ± 1.75	C1 = 21.4 ± 2.03/C2 = 21.73 ± 2.05	60.0	C1 = 66.67/C2 = 73.33	BDJ	C1=RE/C2 = 7BC	12	Depression, Anxiety
Li T/2021 (81)	China/Shanxi	40	40	/	/	0.0	0.0	XYQ	BC	16	BMI, Depression, Anxiety, VOmax, VC
Liu S/2021 (82)	China/Henan	32	24	/	/	/	/	BDJ	BC	8	VC, Step test index
Liu Y/2024 (83)	China/Shandong	31	31	19-21	19-21	48.4	48.4	BDJ	BC	12	BMI, VC
Liu AL/2012 (84)	China/Henan	30	30	/	/	/	/	BDJ	BC	10	VC
Wang ZB/2024 (85)	China/Shanghai	28	26	18.93 ± 0.54	18.96 ± 0.72	0.8	0.9	BDJ	BC	12	Depression, Anxiety
Zhao XD/2018 (86)	China/Beijing	30	30	/	/	/	/	DYYS		12	Depression, Anxiety
Kong YM/2025 (87)	China/Shandong	30	27	18-24	18-24	53.3	51.9	TC	BC	8	Depression, Anxiety
Li M/2015 (10)	China/Fujiang	101	105	20.63 ± 1.03	20.92 ± 1.15	85.1	80.0	BDJ	BC	12	Step test index, VC
Sun J/2025 (88)	China/Shanghai	47	41	18.28 ± 0.97	19.34 ± 1.04	38.3	46.3	TC	BC	16	BMI, VC, SDS, HAMA
Wen N/2024 (12)	China/Shanghai	56	37	19.66 ± 0.79	19.49 ± 0.99	/	/	BDJ	BC	12	BMI, BFR
Xu Y/2025 (89)	China/Shandong	21	22	18.48 ± 0.68	18.27 ± 0.77	0.2	0.1	TC	RE	12	VO2max
Xu ZK/2025 (90)	China/Tianjing	60	60	20.6 ± 2.2	20.3 ± 2.4	33.0	31.8	TC	BC	12	Depression, Anxiety
Ye Y/ (91)	China/Guangdong	61	57	18.9 ± 1.02	18.8 ± 0.99	91.8	94.7	BDJ	BC	12	BMI, Step test index, BFR, VC
Zhang J/2023 (13)	China/Shandong	9	9	24.20 ± 4.07	22.50 ± 5.95	77.8	66.7	TC	BC	8	Depression, Anxiety
Zhang Y/2023 (11)	China/Liaoning	34	39	19.16 ± 1.05	19.23 ± 0.98	/	/	BDJ	BC	12	BMI, VC, Depression, Anxiety
Zheng G/2015 (92)	China/Fujiang	95	103	20.7 ± 1.1	20.6 ± 1.2	65.3	68.9	TC	BC	16	Step test index, VC
Zhao XM/2024 (93)	China/Henan	30	30	19-22	19-22	100.0	100.0	BDJ	RE	16	Depression, Anxiety
Xu K/2026 (94)	China/Shandong	153	50	/	/	/	/	TC	BC	8	Depression, Anxiety
Zhang J/2025 (95)	China/Chongqing	59	C1 = 56/C2 = 58	/	/	66.1	C1 = 71.4/ C2 = 67.2	BDJ	C1=WQX/C2=BC	13	Depression, Anxiety

### 3.3 Results of the methodological quality assessment of the included studies

The risk of bias was appraised for the 69 included studies (**Figure 2**). For the randomization process, 17 studies were judged as at low risk, 47 as having some concerns, and 3 as at high risk. The high risk mainly stemmed from unclear random allocation methods, possible non-random factors in the grouping process, or insufficient reporting of allocation concealment, leading to considerable uncertainty regarding baseline comparability and the authenticity of randomization. Regarding deviations from intended interventions, 58 studies were at low risk, 6 had some concerns, and 3 were at high risk. The high risk was mainly linked to insufficient reporting of intervention implementation procedures, unclear compliance or control of co-interventions, and the possibility that participants or implementors knowing the group allocation could have affected intervention implementation. In terms of missing outcome data, 61 studies were at low risk, 4 had some concerns, and 2 were at high risk. The high risk was mainly due to the presence of dropouts or missing data, no adequate explanation of the reasons for missingness, the proportion of missing data, or the methods for handling them, and the consequent difficulty in determining whether the missingness was related to the true outcomes. For the measurement of the outcome, 35 studies were at low risk, 20 had some concerns, and 12 were at high risk. The high risk mainly involved outcomes measured with subjective scales such as anxiety and depression. Some studies did not implement blinding or did not report whether the outcome assessors were aware of group allocation. Thus, outcome evaluation might have been influenced by expectations regarding the intervention or the measurement process. For selection of the reported result, 12 studies were at low risk, 55 had some concerns, and no studies were at high risk. The overall assessment showed that 46 studies had some concerns, 15 were at high risk, and 6 were at low risk. The methodological quality of the included studies was generally moderate, with the main limitations concentrated in the randomization process, measurement of the outcome, and selection of the reported result.

**Figure 2:**
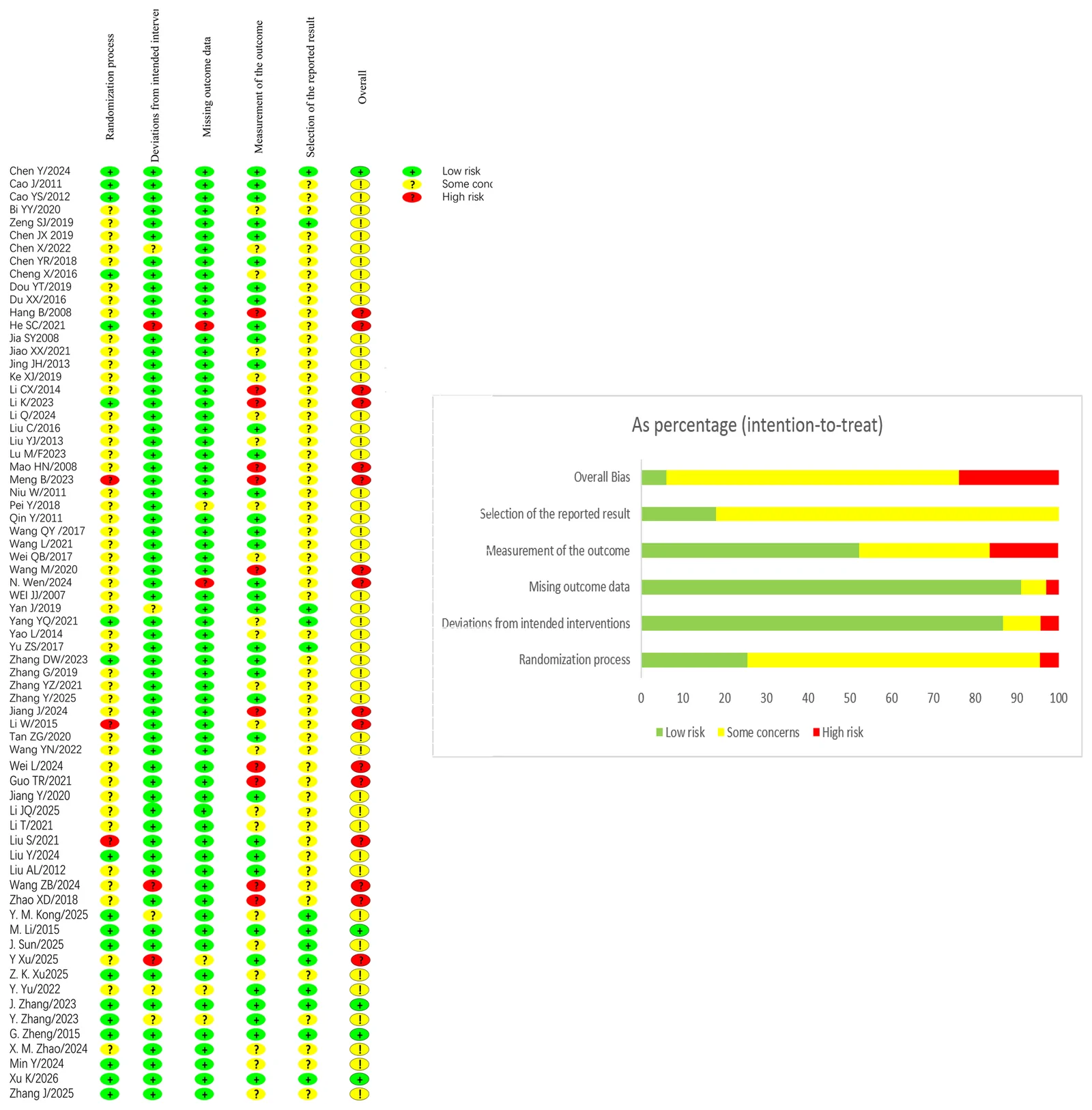
Risk of bias assessment.

### 3.4 Results of network meta-analysis

#### 3.4.1 Consistency check

In the present study, DIC was adopted to compare the consistency model and the inconsistency model (**Supplementary Table S1**) (96, 97). The observed ΔDIC values were all below the threshold: BMI (1.11), BFR (2.7), VC (3.4), VO_2_max (0.5), step test index (0), anxiety (3.54), and depression (0.38). Therefore, the consistency model was selected for all primary network meta-analyses. Bayesian random-effects models showed that, for depression, the posterior median between-study standard deviation was τ = 3.00 (95% CrI: 2.19 to 4.14), and the corresponding between-study variance was τ² = 8.82 (95% CrI: 4.78 to 17.11). For anxiety, the corresponding values were τ = 1.04 (95% CrI: 0.11 to 2.68) and τ² = 1.08 (95% CrI: 0.01 to 7.19). For BMI, the corresponding values were τ = 0.98 (95% CrI: 0.62 to 1.55) and τ² = 0.97 (95% CrI: 0.39 to 2.40). For VC, the corresponding values were τ = 219.95 (95% CrI: 148.62 to 334.75) and τ² = 48,379.21 (95% CrI: 22,086.84 to 112,060.53). For the step test index, the corresponding values were τ = 3.58 (95% CrI: 1.70 to 7.50) and τ² = 12.83 (95% CrI: 2.89 to 56.27). For BFR, the corresponding values were τ = 0.91 (95% CrI: 0.06 to 2.59) and τ² = 0.84 (95% CrI: 0.003 to 6.67). For VO_2_max, the corresponding values were τ = 1.24 (95% CrI: 0.05 to 4.51) and τ² = 1.53 (95% CrI: 0.002 to 20.35). The degree of between-study heterogeneity and the precision of its estimation varied across outcomes. The 95% CrIs for anxiety, step test index, BFR, and VO_2_max were relatively wide, indicating considerable uncertainty in the estimation of heterogeneity parameters. The heterogeneity levels assessed by the I² statistic are detailed in **Supplementary File S3**.

#### 3.4.2 Network plot

The 69 included studies covered 12 distinct interventions. The network structure diagram among distinct interventions is shown in **Figure 3**. In the figure, the thickness of the lines is in direct proportion to the number of studies for each pairwise comparison, and the diameter of the circles is in direct proportion to the number of individuals included in each intervention.

**Figure 3:**
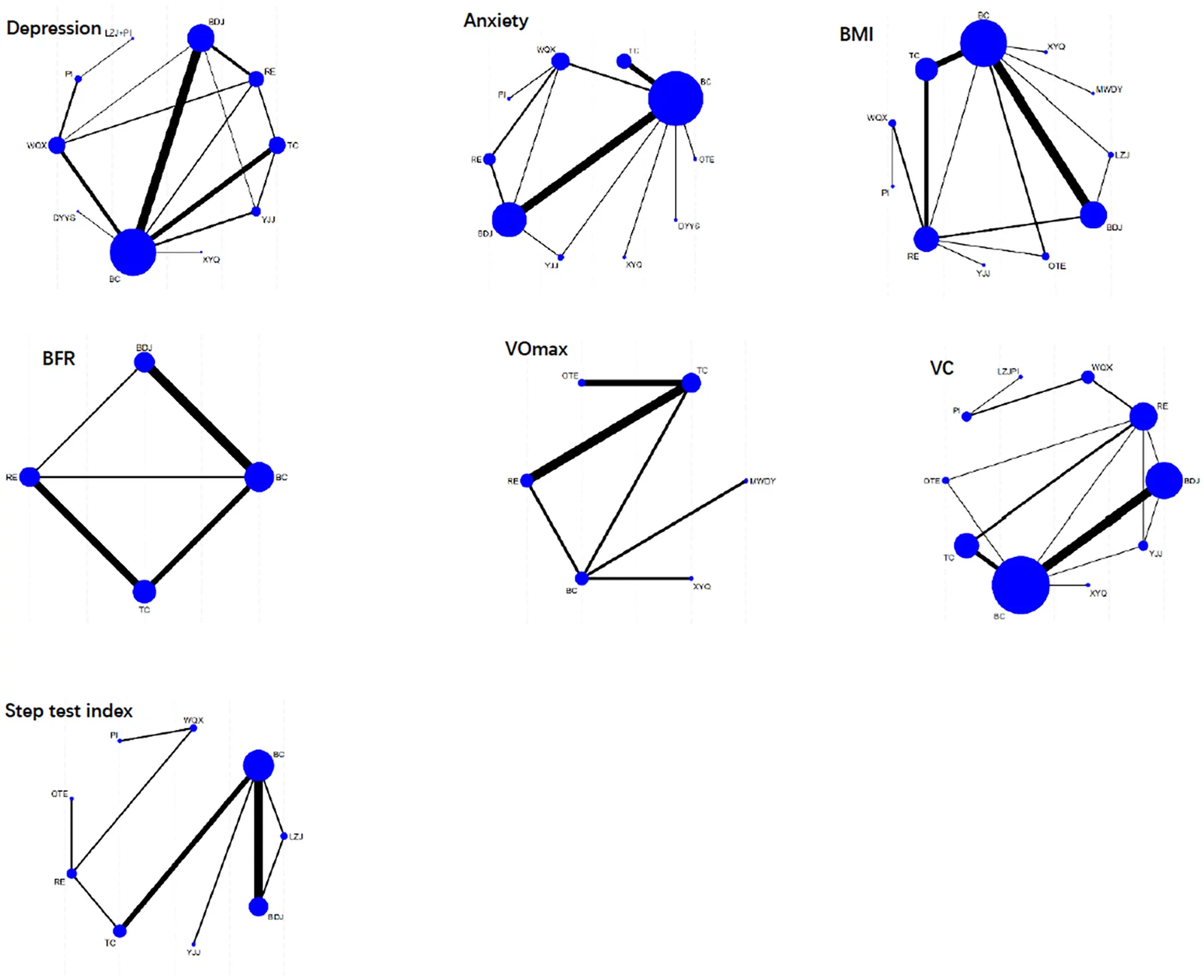
Network plot.

### 3.5 Psychological indicators

Thirty-six studies provided data on depression levels. According to the league table, compared with BC, BDJ (MD = 2.80, 95% CrI: 1.12, 4.64), TC (MD = 5.72, 95% CrI: 3.48, 8.11), and YJJ (MD = 4.55, 95% CrI: 1.80, 7.41) showed significantly greater effects in alleviating depression (**Table 2**). Relative to PI, BDJ (MD = 5.28, 95% CrI: 0.87, 9.88), TC (MD = 8.20, 95% CrI: 3.47, 13.15), WQX (MD = 3.57, 95% CrI: 0.01, 7.20), and YJJ (MD = 7.02, 95% CrI: 1.98, 12.22) also demonstrated superior effects in depression alleviation. Moreover, TC showed significantly greater effects than BDJ (MD = 2.92, 95% CrI: 0.16, 5.67), RE (MD = 4.07, 95% CrI: 1.05, 7.14), and WQX (MD = 4.63, 95% CrI: 1.48, 7.90). Based on the SUCRA results, TC (95.40%), YJJ (85.40%), and BDJ (69.23%) were likely the three most effective exercises (**Supplementary Table S2**; **Figure 4A**).

**Table 2:** League table for depression.

BC	BDJ	DYYS	LZJ _ PI	PI	RE	TC	WQX	XYQ	YJJ
2.8 (1.12, 4.64)	BDJ								
0.25 (-5.81, 6.32)	-2.54 (-8.97, 3.68)	DYYS							
-0.03 (-7.65, 7.5)	-2.83 (-10.63, 4.72)	-0.26 (-9.97, 9.4)	LZJ_PI						
-2.48 (-6.79, 1.84)	-5.28 (-9.88, -0.87)	-2.73 (-10.21, 4.7)	-2.45 (-8.64, 3.81)	PI					
1.65 (-0.78, 4.21)	-1.14 (-3.69, 1.32)	1.41 (-5.11, 8.04)	1.68 (-5.94, 9.52)	4.13 (-0.39, 8.81)	RE				
5.72 (3.48, 8.11)	2.92 (0.16, 5.67)	5.47 (-0.93, 12.05)	5.73 (-2, 13.78)	8.2 (3.47, 13.15)	4.07 (1.05, 7.14)	TC			
1.1 (-1.26, 3.51)	-1.7 (-4.49, 0.95)	0.85 (-5.66, 7.41)	1.13 (-6.02, 8.37)	3.57 (0.01, 7.2)	-0.56 (-3.46, 2.26)	-4.63 (-7.9, -1.48)	WQX		
0.25 (-5.79, 6.29)	-2.54 (-8.95, 3.65)	0 (-8.62, 8.56)	0.28 (-9.42, 9.99)	2.73 (-4.73, 10.18)	-1.4 (-8.01, 5.08)	-5.47 (-12.05, 0.9)	-0.84 (-7.39, 5.61)	XYQ	
4.55 (1.8, 7.41)	1.75 (-1.39, 4.82)	4.3 (-2.32, 11.03)	4.58 (-3.34, 12.72)	7.02 (1.98, 12.22)	2.89 (-0.7, 6.5)	-1.17 (-4.36, 1.96)	3.45 (-0.14, 7.12)	4.3 (-2.31, 11.04)	YJJ

**Figure 4:**
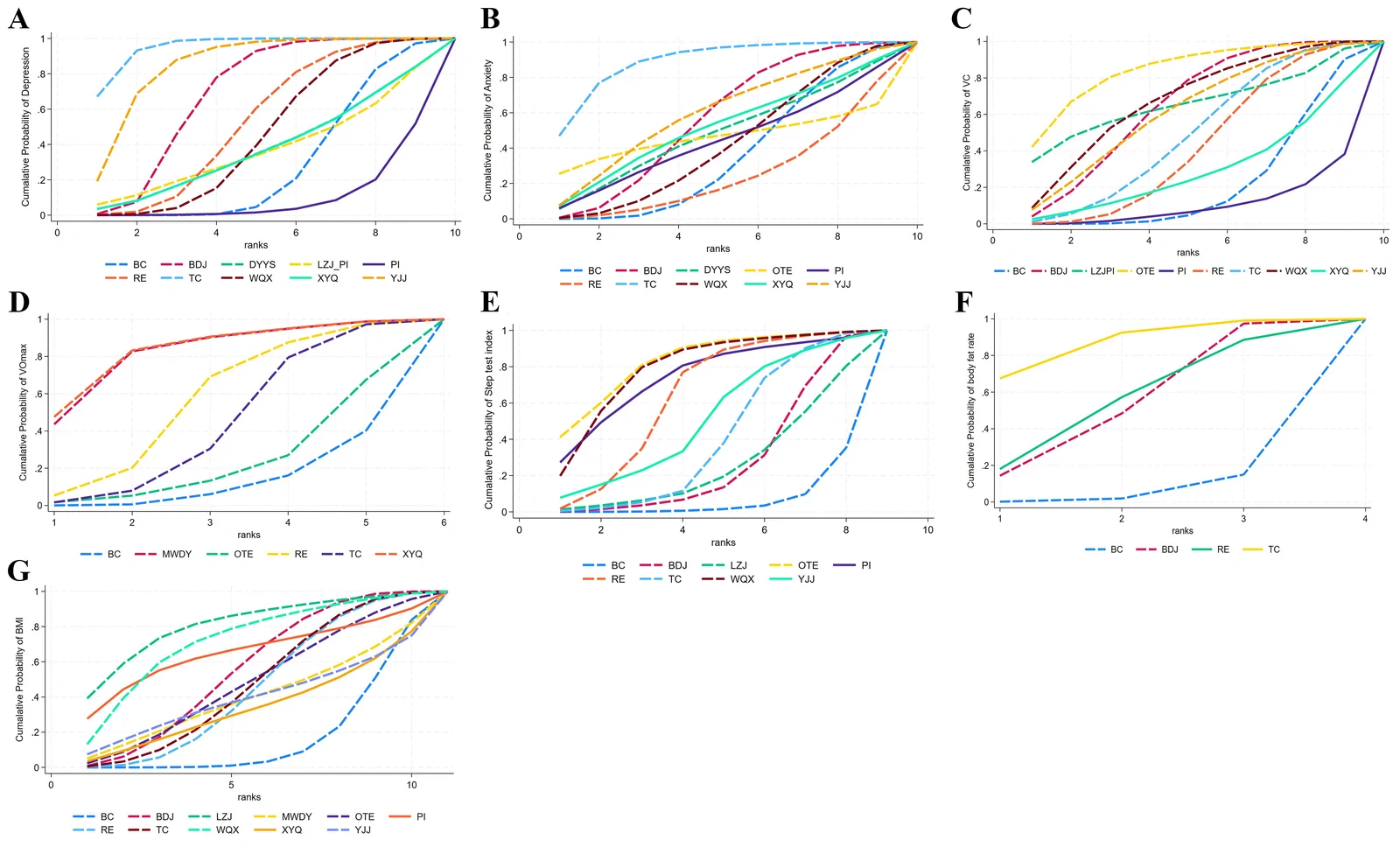
Cumulative probability ranking for **(A)** depression, **(B)** anxiety, **(C)** vital capacity, **(D)** VO2 max, **(E)** step test index, **(F)** body fat rate, and **(G)** BMI.

Twenty-five studies reported on anxiety levels. According to the league table, TC showed a significantly greater effect on anxiety alleviation than BC (MD = 1.70, 95% CrI: 0.12, 4.35), whereas the 95% CrIs for the remaining pairwise comparisons generally included zero (**Table 3**). These findings suggested that although TC demonstrated a significant advantage over BC, the relative effects among most active interventions remained uncertain. As per the SUCRA values, TC (88.82%), YJJ (59.78%), and BDJ (56.78%) were potentially the three most optimal exercises (**Supplementary Table S2**; **Figure 4B**).

**Table 3:** League table for anxiety.

BC	BDJ	DYYS	OTE	PI	RE	TC	WQX	XYQ	YJJ
0.29 (-0.45, 1.64)	BDJ								
0.16 (-2.76, 3.09)	-0.1 (-3.55, 2.66)	DYYS							
0.15 (-5.42, 5.64)	-0.25 (-5.98, 5.28)	-0.02 (-6.19, 6.11)	OTE						
0.07 (-3.49, 3.3)	-0.2 (-4.12, 2.72)	-0.09 (-4.76, 4.19)	-0.13 (-6.56, 6.21)	PI					
-0.28 (-2.91, 1.46)	-0.62 (-3.46, 0.87)	-0.42 (-4.56, 2.75)	-0.57 (-6.6, 5.23)	-0.32 (-4.07, 2.78)	RE				
1.7 (0.12, 4.35)	1.21 (-0.17, 4.01)	1.25 (-0.96, 5.88)	1.55 (-4.14, 7.96)	1.37 (-1.12, 6.58)	2 (0, 6.19)	TC			
0.06 (-1.87, 1.63)	-0.24 (-2.51, 1.09)	-0.1 (-3.7, 3.12)	-0.14 (-5.93, 5.6)	-0.01 (-2.91, 2.9)	0.31 (-1.18, 2.43)	-1.58 (-5.25, 0.14)	WQX		
0.23 (-2.68, 3.16)	-0.02 (-3.46, 2.74)	0.07 (-4.03, 4.22)	0.09 (-6.04, 6.27)	0.16 (-4.11, 4.83)	0.49 (-2.66, 4.64)	-1.18 (-5.81, 1.02)	0.17 (-3.06, 3.76)	XYQ	
0.39 (-1.43, 3.28)	0.11 (-2.07, 2.73)	0.21 (-2.97, 4.53)	0.43 (-5.36, 6.61)	0.32 (-3.04, 5.11)	0.73 (-1.54, 4.82)	-0.89 (-4.57, 1.21)	0.36 (-1.86, 3.92)	0.14 (-3.06, 4.44)	YJJ

### 3.6 Cardiopulmonary function

Twenty-eight studies examined VC. The league table revealed that BDJ significantly improved VC in comparison with BC (MD= −243.96, 95% CrI: −398.66, −85.79), and WQX also exhibited a significant advantage over PI (MD= −500.67, 95% CrI: −901.22, −101.05). No other pairwise comparisons demonstrated statistically significant differences (**Table 4**). The SUCRA results indicated that OTE (84.53%), WQX (67.72%), and LZJ+PI (65.82%) were likely the three most effective exercises (**Supplementary Table S2**; **Figure 4C**).

**Table 4:** League table for vital capacity.

BC	BDJ	LZJ _ PI	OTE	PI	RE	C	WQX	XYQ	YJJ
-243.96 (-398.66, -85.79)	BDJ								
-333.86 (-1220.25, 565.12)	-89.54 (-983.59, 812.06)	LZJPI							
-445.19 (-874.44, 3.24)	-201.25 (-647.64, 262.33)	-112.19 (-1059.84, 837.27)	OTE						
216.04 (-382.28, 827.41)	459.56 (-142.97, 1077.48)	549.76 (-109.22, 1207.4)	662.22 (-26.34, 1335.93)	PI					
-125.96 (-397.24, 153.25)	117.62 (-167.77, 410.51)	208.62 (-647.04, 1052.84)	320.29 (-103.99, 726.42)	-342.39 (-884.09, 194.45)	RE				
-157.21 (-379.91, 73.29)	86.63 (-175.72, 356.04)	177.22 (-718.33, 1064.58)	288.17 (-180.92, 741.36)	-373.98 (-982.69, 228.27)	-31.36 (-311.16, 248.27)	TC			
-284.49 (-729.21, 175.82)	-40.24 (-495.51, 427.37)	49.4 (-721.05, 819.92)	161.66 (-394.08, 705.69)	-500.67 (-901.22, -101.05)	-158.33 (-515.94, 205.21)	-126.92 (-578.62, 330.49)	WQX		
-0.73 (-462.41, 458.99)	243.52 (-246.05, 728.05)	333.86 (-683.57, 1326.72)	445.03 (-206.19, 1068.39)	-216.09 (-987.03, 533.95)	125.24 (-420.08, 657.11)	156.79 (-363.17, 663.35)	283.69 (-372, 919.29)	XYQ	
-238.2 (-590.49, 119.92)	5.6 (-355.56, 371.7)	94.46 (-831.77, 1020.67)	207.48 (-326.94, 723.31)	-455.38 (-1105.79, 192.6)	-112.3 (-481.63, 253.68)	-81.39 (-476.86, 313.48)	45.71 (-471.36, 558.08)	-237.84 (-814.46, 347.33)	YJJ

Six studies presented data on VO_2_max. The league table demonstrated that both MWDY (MD= −5.09, 95% CrI: −9.71, −0.37) and Xingyiquan (XYQ) (MD= −5.18, 95% CrI: −9.92, −0.42) were substantially superior to BC. No other pairwise differences were statistically significant (**Table 5**). The SUCRA results suggested that XYQ (82.89%), MWDY (82.07%), and RE (56.12%) were likely the three best exercises (**Supplementary Table S2** and **Figure 4D**).

**Table 5:** League table for VO2 max.

BC	MWDY	OTE	RE	TC	XYQ
-5.09 (-9.71, -0.37)	MWDY				
-0.76 (-6.26, 4.73)	4.33 (-2.96, 11.5)	OTE			
-2.62 (-7.04, 1.76)	2.48 (-4, 8.78)	-1.84 (-6.5, 2.71)	RE		
-1.85 (-6.27, 2.51)	3.22 (-3.21, 9.55)	-1.09 (-4.43, 2.24)	0.74 (-2.41, 3.98)	TC	
-5.18 (-9.92, -0.42)	-0.1 (-6.77, 6.48)	-4.44 (-11.67, 2.87)	-2.59 (-9.01, 3.93)	-3.34 (-9.72, 3.12)	XYQ

Thirteen studies assessed the step test index. The league table showed that only OTE exhibited a significant advantage over BC (MD= −14.25, 95% CrI: −28.28, −0.16), while all other pairwise comparisons failed to reach statistical significance (**Table 6**). According to the SUCRA values, OTE (82.52%), XYQ (78.81%), and PI (73.88%) were potentially the three most optimal exercises (**Supplementary Table S2** and **Figures 4E**).

**Table 6:** League table for step test index.

BC	BDJ	LZJ	OTE	PI	RE	TC	WQX	YJJ
-3.02 (-6.97, 1.05)	BDJ							
-2.68 (-10.62, 5.37)	0.35 (-7.7, 8.29)	LZJ						
-14.25 (-28.28, -0.16)	-11.22 (-25.88, 3.42)	-11.59 (-27.7, 4.59)	OTE					
-12.97 (-29.66, 3.88)	-9.97 (-27.19, 7.31)	-10.29 (-28.91, 8.32)	1.29 (-14.22, 16.78)	PI				
-10.43 (-21.39, 0.69)	-7.41 (-19.13, 4.33)	-7.77 (-21.39, 5.9)	3.83 (-4.98, 12.64)	2.53 (-10.12, 15.25)	RE			
-5.07 (-10.42, 0.34)	-2.06 (-8.76, 4.61)	-2.41 (-12.06, 7.22)	9.16 (-3.86, 22.24)	7.92 (-8.07, 23.81)	5.36 (-4.23, 14.97)	TC		
-13.38 (-27.34, 0.69)	-10.37 (-24.93, 4.21)	-10.71 (-26.86, 5.53)	0.87 (-11.54, 13.32)	-0.42 (-9.65, 8.85)	-2.94 (-11.7, 5.79)	-8.32 (-21.26, 4.61)	WQX	
-6.88 (-15.88, 2.19)	-3.85 (-13.73, 5.94)	-4.2 (-16.22, 7.84)	7.38 (-9.34, 24.16)	6.13 (-12.97, 25.12)	3.56 (-10.7, 17.84)	-1.8 (-12.27, 8.73)	6.52 (-10.18, 23.17)	YJJ

### 3.7 Body composition

Thirteen studies documented BFR. The league table reflected that only TC was significantly superior to BC (MD = 2.47, 95% CrI: 0.22, 4.06). No other pairwise comparisons were statistically significant (**Table 7**). The SUCRA values indicated that TC (86.35%), RE (54.60%), and BDJ (53.39%) were likely the three most optimum exercises (**Supplementary Table S2** and **Figure 4F**).

**Table 7:** League table for body fat rate.

BC	BDJ	RE	TC
1.4 (-0.11, 2.91)	BDJ		
1.53 (-1.12, 3.96)	0.14 (-2.78, 2.8)	RE	
2.47 (0.22, 4.06)	1.09 (-1.67, 3.08)	0.9 (-1.46, 3.14)	TC

Thirty studies provided data on BMI. According to the league table, only BDJ showed a significant advantage over BC (MD = 0.88, 95% CrI: 0.14, 1.62). No other pairwise comparisons reached statistical significance (**Table 8**). The SUCRA results uncovered that LZJ (81.27%), WQX (72.38%), and PI (65.44%) were likely the three best exercises (**Supplementary Table S2** and **Figure 4G**).

**Table 8:** League table for BMI.

BC	BDJ	LZJ	MWDY	OTE	PI	RE	TC	WQX	XYQ	YJJ
0.88 (0.14, 1.62)	BDJ									
1.96 (-0.17, 4.11)	1.09 (-1.06, 3.22)	LZJ								
0.46 (-1.77, 2.68)	-0.41 (-2.76, 1.93)	-1.5 (-4.6, 1.59)	MWDY							
0.73 (-0.69, 2.12)	-0.15 (-1.72, 1.38)	-1.24 (-3.8, 1.3)	0.26 (-2.38, 2.87)	OTE						
1.49 (-1.91, 4.88)	0.62 (-2.81, 4.04)	-0.47 (-4.47, 3.5)	1.03 (-3.02, 5.1)	0.76 (-2.8, 4.35)	PI					
0.67 (-0.39, 1.71)	-0.21 (-1.34, 0.9)	-1.29 (-3.64, 1.04)	0.21 (-2.26, 2.66)	-0.06 (-1.55, 1.46)	-0.83 (-4.04, 2.4)	RE				
0.7 (-0.17, 1.61)	-0.18 (-1.25, 0.93)	-1.26 (-3.55, 1.05)	0.24 (-2.15, 2.65)	-0.03 (-1.56, 1.58)	-0.8 (-4.16, 2.59)	0.03 (-0.97, 1.08)	TC			
1.51 (-0.56, 3.57)	0.63 (-1.47, 2.72)	-0.45 (-3.39, 2.48)	1.05 (-2, 4.08)	0.78 (-1.53, 3.13)	0.01 (-2.69, 2.72)	0.84 (-0.93, 2.62)	0.81 (-1.25, 2.84)	WQX		
0.28 (-1.92, 2.48)	-0.60 (-2.92, 1.72)	-1.68 (-4.76, 1.38)	-0.18 (-3.3, 2.95)	-0.45 (-3.05, 2.19)	-1.21 (-5.26, 2.82)	-0.39 (-2.82, 2.06)	-0.42 (-2.81, 1.94)	-1.23 (-4.25, 1.79)	XYQ	
0.4 (-2.46, 3.29)	-0.47 (-3.37, 2.44)	-1.56 (-5.11, 2.00)	-0.06 (-3.71, 3.58)	-0.32 (-3.38, 2.77)	-1.09 (-5.28, 3.11)	-0.26 (-2.94, 2.43)	-0.3 (-3.17, 2.57)	-1.1 (-4.31, 2.12)	0.12 (-3.49, 3.77)	YJJ

### 3.8 Publication bias

Comparison-adjusted funnel plots were applied to evaluate potential small-study effects or publication bias for each outcome indicator. The funnel plots for depression, anxiety, VC, VO_2_max, step test index, BFR, and BMI were generally symmetric in distribution, and no significant small-study effects or publication bias were observed (**Figure 5**).

**Figure 5:**
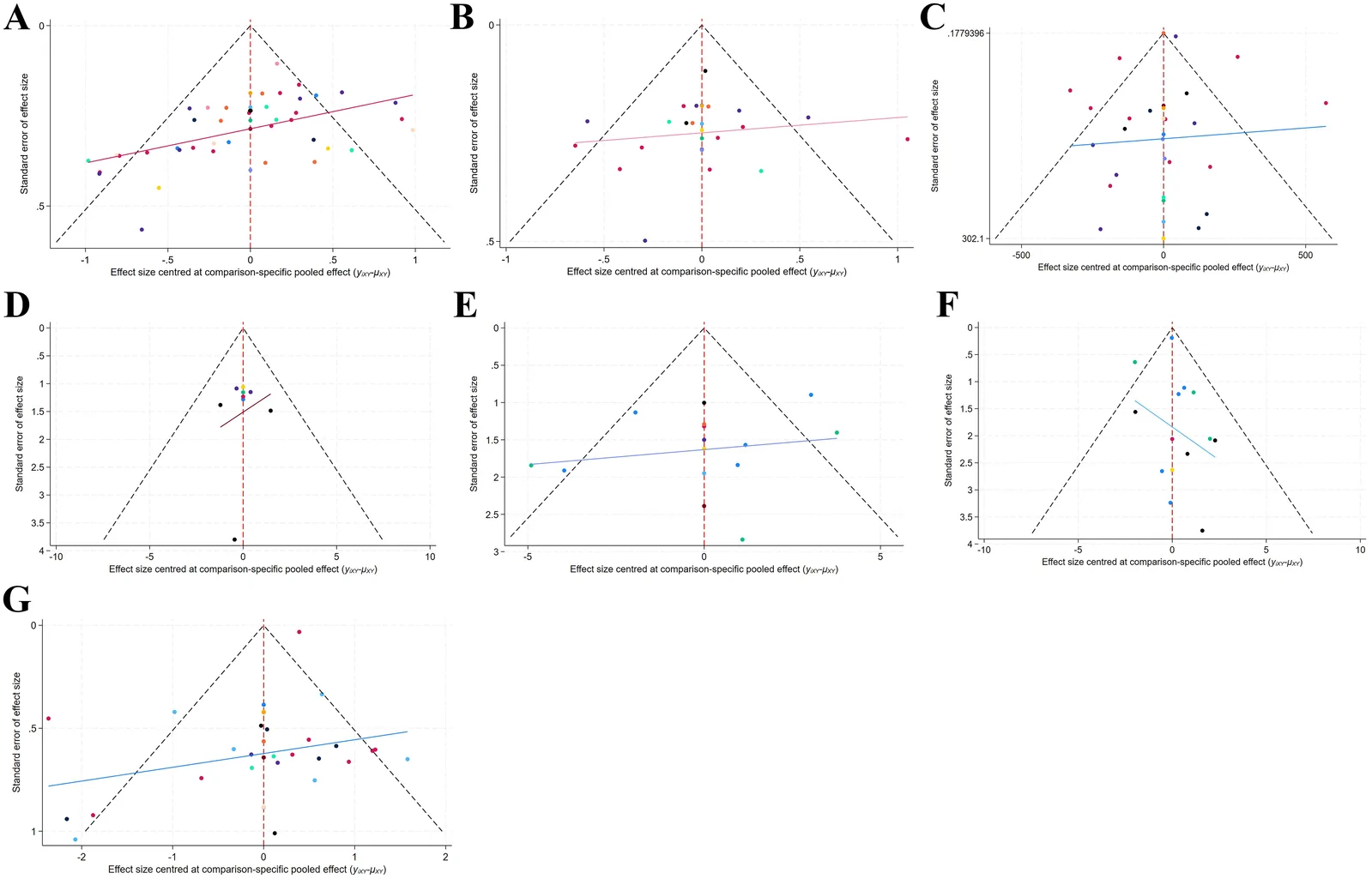
Publication bias assessment for **(A)** depression, **(B)** anxiety, **(C)** vital capacity, **(D)** VO2 max, **(E)** step test index, **(F)** body fat rate, and **(G)** BMI.

## 4 Discussion

This study systematically compared the relative effects of multiple traditional Chinese fitness exercises on the multidimensional outcomes of mental health, body composition, and cardiorespiratory function among college students. Totally 69 RCTs involving 6,065 college students were included. The results indicate that traditional Chinese fitness exercises have the potential to yield multidimensional benefits for the physical and mental health of college students, but the superior outcomes vary across different exercises. For depression, TC, YJJ, and BDJ showed the most prominent ranking advantages, with TC demonstrating significant superiority over BC and several active interventions. For anxiety, TC showed a significant advantage over BC, while the relative effects among most active interventions remained uncertain. According to the SUCRA rankings, TC, YJJ, and BDJ ranked highest.

Regarding body composition, TC showed a clearer advantage in BFR, while BDJ exhibited a more pronounced advantage in BMI. In terms of cardiorespiratory function, BDJ and WQX presented certain advantages in VC. MWDY and XYQ showed some superiority in VO_2_max. OTE along with XYQ demonstrated certain strengths in the step test index. Overall, the core finding of this study is not to identify a single optimal exercise for all outcomes, but rather to reveal an outcome-specific pattern of advantages across different traditional Chinese fitness exercises in improving the physical and mental health of college students.

In terms of mental health, the present study found that TC, YJJ, and BDJ were particularly effective in alleviating depression, which is generally consistent with previous meta-analyses of qigong exercises and traditional Chinese health maintenance exercises among college students (19, 98). TC showed the highest SUCRA value for depression (95.40%), followed by YJJ (85.40%) and BDJ (69.23%). TC may exert its effects through the pathway of “slow continuous movements – stable breathing rhythm – enhanced prefrontal cortex control – reduced processing of negative emotions”. Its weight shifting, postural control, and coordinated breathing continuously engage proprioception and executive control, thereby strengthening the prefrontal cortex’s ability to regulate negative emotional stimuli (13, 99). YJJ operates more through a mechanism chain of “ muscle-bone stretching – chest opening and closing – enhanced interoceptive feedback – increased self-efficacy in emotion regulation”. In this chain, the integration of stretching, breathing, and concentration shifts attention from rumination to bodily awareness, reducing tension and low-activation experiences (14, 100). BDJ follows a comprehensive pathway of “standardized movements – mild whole-body load – regulation of the autonomic nervous system and the hypothalamic-pituitary-adrenal (HPA) axis – enhanced self-efficacy”. Its movements are easy to learn and the load is moderate, facilitating sustained practice. Previous studies have also supported its positive effects on anxiety, depression, and overall mental health (101, 102). For anxiety, TC showed a statistically significant advantage over BC and had the highest SUCRA value (88.82%), followed by YJJ (59.78%) and BDJ (56.78%). This finding is broadly consistent with recent evidence showing that TC can reduce anxiety symptoms in college students (103) and with a network meta-analysis in a broader adult population in which TC also ranked highly for anxiety reduction (104). Nevertheless, the relatively wide CrIs observed across several comparisons indicate limited precision in the current network and suggest that additional direct head-to-head evidence is required. The remaining uncertainty may also be influenced by the temporal and situational variability of anxiety symptoms (105) and by differences in the psychometric properties of commonly used anxiety scales among Chinese university students (106). Taken together, the current findings support TC as a promising exercise modality for alleviating anxiety in college students. However, the available evidence remains insufficient to conclude that it is superior to most other traditional Chinese fitness exercises.

For cardiorespiratory function, the advantageous effects on VC, VO_2_max, and step test index are not consistent. This suggests that the impact of traditional fitness exercises on cardiorespiratory function is indicator-specific. VC primarily reflects ventilation capacity, chest mobility, and respiratory system function, and is more easily influenced by breathing coordination, chest opening and closing, and respiratory muscle involvement (107). In this study, WQX and BDJ showed advantages in VC, which may correspond to the pathway of “limb stretching – chest opening and closing – respiratory muscle involvement – improved ventilation capacity”. BDJ has been reported to improve physical fitness indicators like VC, flexibility, and body composition in college students (91), while WQX, characterized by trunk rotations, lower-limb support, and whole-body coordination, is more commonly applied in respiratory system diseases (108). VO_2_max reflects the body’s comprehensive capacity to take up, transport, and utilize oxygen, and is an important indicator of cardiorespiratory fitness (109). XYQ and MWDY showed advantages in this indicator, which may be associated with continuous dynamic movements, lower-limb loading, and enhanced circulatory transport capacity (18, 31). The step test index is simultaneously influenced by cardiorespiratory endurance, lower-limb muscular endurance, and post-exercise recovery ability. Therefore, the high ranking probabilities observed for OTE and XYQ may be related to improvements in overall exercise load and recovery capacity. Overall, the differentiation of cardiorespiratory indicators among different exercises does not reflect contradictory results but rather a reasonable manifestation of differences in movement structure, breathing involvement, lower-limb loading, and sustained exercise duration (16).

Regarding body composition, the advantageous effects on BFR and BMI are not entirely consistent. This indicates that these two should not be regarded as the same outcome. BFR more directly reflects the proportion of adipose tissue, whereas BMI only represents the ratio of body weight to height and cannot differentiate among fat mass, lean body mass, and muscle mass (110). In this study, TC showed a significant advantage over BC in BFR, while BDJ exhibited a significant advantage over BC in BMI. These findings suggest that the relative effects of different exercises may vary according to the specific body-composition indicator assessed. The favorable ranking of TC for BFR may be related to the mechanism chain of “low-posture control – continuous weight shifting – increased static and dynamic lower-limb loading – regulation of fat metabolism. Previous systematic reviews have shown that TC can improve physical fitness indicators like BMI, BFR, VC, and flexibility (111, 112). The improvement in BMI by BDJ may be explained by the pathway of “standardized whole-body movements – mild and sustained loading – improved exercise adherence – enhanced weight management”. An RCT for college students has also demonstrated its ability to improve body weight status, BMI, BFR, and fat mass (12). LZJ and WQX ranked high in terms of BMI improvement, but pairwise comparisons were not statistically significant. This finding should be interpreted as an indication of a trend. Furthermore, the effects of WQX on reducing metabolic syndrome-related risk factors also suggest its potential value in body weight and metabolic regulation (17).

In conclusion, traditional Chinese fitness exercises offer multidimensional potential benefits for the mental health, body composition, and cardiorespiratory function of college students. However, the advantages of different exercises are not concentrated on a single outcome. TC, YJJ, and BDJ show relatively prominent effects in alleviating depression. For anxiety, TC had the highest ranking probability and showed a statistically significant advantage over BC. TC and BDJ demonstrate certain advantages in BFR and BMI, respectively; BDJ, WQX, MWDY, XYQ, and OTE exhibit differentiated benefits across various cardiorespiratory function indicators. It should be emphasized that, except for some outcomes with clear pairwise comparative advantages, other results are still primarily based on SUCRA values and suggestive evidence of a trend and cannot be directly considered as definitive recommendations. Future researchers should incorporate more pre-registered, multicenter, large-sample RCTs, strengthen direct comparisons among different exercises, standardize outcome indicators and measurement methods, and systematically report intervention dosage, adherence, adverse events, and follow-up results. Overall, traditional Chinese fitness exercises can serve as important candidate strategies for promoting the physical and mental health of college students. Nevertheless, we should not pursue a single optimal exercise but rather make an outcome-oriented, precise selection based on specific health goals.

## Data Availability

The original contributions presented in the study are included in the article/**Supplementary Material**. Further inquiries can be directed to the corresponding author.
