# Supplementary material for: Network meta-analysis of the effects of traditional Chinese fitness exercises on mental health and physical benefits among college students
Source: Front Psychiatry. 2026 Oct 9;17:1921373. doi: 10.3389/fpsyt.2026.1921373 (PMC13690765; doi:10.3389/fpsyt.2026.1921373)
Supplement: Supplementary file 1 [file SupplementaryFile1.docx]

**Supplementary materials**

**File** **S1** PRISMA+NMA+checklist

**PRISMA NMA Checklist of Items to Include When Reporting A Systematic Review Involving a Network Meta-analysis**

| **Section/Topic** | **Item #** | **Checklist Item** | **Reported in section or paragraph** |
| --- | --- | --- | --- |
| **TITLE** |  |  |  |
| Title | 1 | Identify the report as a systematic review *incorporating a network meta-analysis (or related form of meta-analysis).* | Title |
|  |  |  |  |
| **ABSTRACT** |  |  |  |
| Structured summary | 2 | Provide a structured summary including, as applicable:  **Background:** main objectives  **Methods:** data sources; study eligibility criteria, participants, and interventions; study appraisal; and *synthesis methods, such as network meta-analysis.*  **Results:** number of studies and participants identified; summary estimates with corresponding confidence/credible intervals; *treatment rankings may also be discussed. Authors may choose to summarize pairwise comparisons against a chosen treatment included in their analyses for brevity.*  **Discussion/Conclusions:** limitations; conclusions and implications of findings.  **Other:** primary source of funding; systematic review registration number with registry name. | Abstract |
|  |  |  |  |
| **INTRODUCTION** |  |  |  |
| Rationale | 3 | Describe the rationale for the review in the context of what is already known*, including mention of why a network meta-analysis has been conducted.* | Introduction, paragraphs 2–3 |
| Objectives | 4 | Provide an explicit statement of questions being addressed, with reference to participants, interventions, comparisons, outcomes, and study design (PICOS). | Introduction; Methods 2.2 |
|  |  |  |  |
| **METHODS** |  |  |  |
| Protocol and registration | 5 | Indicate whether a review protocol exists and if and where it can be accessed (e.g., Web address); and, if available, provide registration information, including registration number. | Methods, opening paragraph |
| Eligibility criteria | 6 | Specify study characteristics (e.g., PICOS, length of follow-up) and report characteristics (e.g., years considered, language, publication status) used as criteria for eligibility, giving rationale. *Clearly describe eligible treatments included in the treatment network, and note whether any have been clustered or merged into the same node (with justification).* | Methods, Section 2.2 |
| Information sources | 7 | Describe all information sources (e.g., databases with dates of coverage, contact with study authors to identify additional studies) in the search and date last searched. | Methods, Section 2.1 |
| Search | 8 | Present full electronic search strategy for at least one database, including any limits used, such that it could be repeated. | Methods, Section 2.1; File S2 |
| Study selection | 9 | State the process for selecting studies (i.e., screening, eligibility, included in systematic review, and, if applicable, included in the meta-analysis). | Methods, Section 2.3, paragraph 1 |
| Data collection process | 10 | Describe method of data extraction from reports (e.g., piloted forms, independently, in duplicate) and any processes for obtaining and confirming data from investigators. | Section 2.3, paragraph 2 |
| Data items | 11 | List and define all variables for which data were sought (e.g., PICOS, funding sources) and any assumptions and simplifications made. | Methods, Sections 2.2–2.3 |
| **Geometry of the network** | **S1** | Describe methods used to explore the geometry of the treatment network under study and potential biases related to it. This should include how the evidence base has been graphically summarized for presentation, and what characteristics were compiled and used to describe the evidence base to readers. | Methods, Section 2.5; Results, Section 3.4.2 |
| Risk of bias within individual studies | 12 | Describe methods used for assessing risk of bias of individual studies (including specification of whether this was done at the study or outcome level), and how this information is to be used in any data synthesis. | Methods, Section 2.4 |
| Summary measures | 13 | State the principal summary measures (e.g., risk ratio, difference in means). *Also describe the use of additional summary measures assessed, such as treatment rankings and surface under the cumulative ranking curve (SUCRA) values, as well as modified approaches used to present summary findings from meta-analyses.* | Methods, Section 2.5 |
| Planned methods of analysis | 14 | Describe the methods of handling data and combining results of studies for each network meta-analysis. This should include, but not be limited to:   - *Handling of multi-arm trials;* - *Selection of variance structure;* - *Selection of prior distributions in Bayesian analyses; and* - *Assessment of model fit.* | Methods, Sections 2.5; Results, Section 3.4.1 |
| **Assessment of Inconsistency** | **S2** | Describe the statistical methods used to evaluate the agreement of direct and indirect evidence in the treatment network(s) studied. Describe efforts taken to address its presence when found. | Methods, Section 2.5 |
| Risk of bias across studies | 15 | Specify any assessment of risk of bias that may affect the cumulative evidence (e.g., publication bias, selective reporting within studies). | Methods, Section 2.5, final sentence |
| Additional analyses | 16 | Describe methods of additional analyses if done, indicating which were pre-specified. This may include, but not be limited to, the following:   - Sensitivity or subgroup analyses; - Meta-regression analyses; - *Alternative formulations of the treatment network; and* - *Use of alternative prior distributions for Bayesian analyses (if applicable).* | Not reported |
|  |  |  |  |
| **RESULTS†** |  |  |  |
| Study selection | 17 | Give numbers of studies screened, assessed for eligibility, and included in the review, with reasons for exclusions at each stage, ideally with a flow diagram. | Results, Section 3.1; Figure 1 |
| **Presentation of network structure** | **S3** | Provide a network graph of the included studies to enable visualization of the geometry of the treatment network. | Results, Section 3.4.2; Figure 3 |
| **Summary of network geometry** | **S4** | Provide a brief overview of characteristics of the treatment network. This may include commentary on the abundance of trials and randomized patients for the different interventions and pairwise comparisons in the network, gaps of evidence in the treatment network, and potential biases reflected by the network structure. | Results, Sections 3.2 and 3.4.2; Figure 3 |
| Study characteristics | 18 | For each study, present characteristics for which data were extracted (e.g., study size, PICOS, follow-up period) and provide the citations. | Results, Section 3.2; Table 1 |
| Risk of bias within studies | 19 | Present data on risk of bias of each study and, if available, any outcome level assessment. | Results, Section 3.3; Figure 2 |
| Results of individual studies | 20 | For all outcomes considered (benefits or harms), present, for each study: 1) simple summary data for each intervention group, and 2) effect estimates and confidence intervals. *Modified approaches may be needed to deal with information from larger networks.* | File S3, subsections (1)–(7) |
| Synthesis of results | 21 | Present results of each meta-analysis done, including confidence/credible intervals. *In larger networks, authors may focus on comparisons versus a particular comparator (e.g. placebo or standard care), with full findings presented in an appendix. League tables and forest plots may be considered to summarize pairwise comparisons.* If additional summary measures were explored (such as treatment rankings), these should also be presented. | Results, Sections 3.4.1 and 3.5–3.7; Tables 2–8; Figure 4; File S3; Table S2 |
| **Exploration for inconsistency** | **S5** | Describe results from investigations of inconsistency. This may include such information as measures of model fit to compare consistency and inconsistency models, *P* values from statistical tests, or summary of inconsistency estimates from different parts of the treatment network. | Results, Section 3.4.1; Table S1 |
| Risk of bias across studies | 22 | Present results of any assessment of risk of bias across studies for the evidence base being studied. | Results, Section 3.8; Figure 5 |
| Results of additional analyses | 23 | Give results of additional analyses, if done (e.g., sensitivity or subgroup analyses, meta-regression analyses*, alternative network geometries studied, alternative choice of prior distributions for Bayesian analyses,* and so forth). | Not reported |
|  |  |  |  |
| **DISCUSSION** |  |  |  |
| Summary of evidence | 24 | Summarize the main findings, including the strength of evidence for each main outcome; consider their relevance to key groups (e.g., healthcare providers, users, and policy-makers). | Discussion, Section 4, paragraphs 1–6 |
| Limitations | 25 | Discuss limitations at study and outcome level (e.g., risk of bias), and at review level (e.g., incomplete retrieval of identified research, reporting bias). *Comment on the validity of the assumptions, such as transitivity and consistency. Comment on any concerns regarding network geometry (e.g., avoidance of certain comparisons).* | Section 4, paragraphs 3, 5–6; Section 3.3 |
| Conclusions | 26 | Provide a general interpretation of the results in the context of other evidence, and implications for future research. | Discussion, Section 4, paragraph 6 |
|  |  |  |  |
| **FUNDING** |  |  |  |
| Funding | 27 | Describe sources of funding for the systematic review and other support (e.g., supply of data); role of funders for the systematic review. This should also include information regarding whether funding has been received from manufacturers of treatments in the network and/or whether some of the authors are content experts with professional conflicts of interest that could affect use of treatments in the network. | Funding statement |

PICOS = population, intervention, comparators, outcomes, study design.

* Text in italics indicateS wording specific to reporting of network meta-analyses that has been added to guidance from the PRISMA statement.

† Authors may wish to plan for use of appendices to present all relevant information in full detail for items in this section.

**File S2** Literature retrieval strategy

(1) Search History of PubMed

| Search number | Query | Results |
| --- | --- | --- |
| 1 | "Tai Ji"[Mesh] | 1,745 |
| 2 | "Qigong"[Mesh] | 462 |
| 3 | "undergraduate"[Title/Abstract] OR "postgraduate"[Title/Abstract] OR "freshman"[Title/Abstract] OR "student*"[Title/Abstract] | 464,848 |
| 4 | "Ba Duan Jin"[Title/Abstract] OR "Baduanjin"[Title/Abstract] OR "Ch i Kung"[Title/Abstract] OR "chi kung"[Title/Abstract] OR "chigung"[Title/Abstract] OR "Chinese traditional exercise"[Title/Abstract] OR "Dao Yin"[Title/Abstract] OR "Daoyin"[Title/Abstract] OR "Five Animal Frolics"[Title/Abstract] OR "health qigong"[Title/Abstract] OR "Liu Zi Jue"[Title/Abstract] OR "Liuzijue"[Title/Abstract] OR "mind body exercise"[Title/Abstract] OR "qi gong"[Title/Abstract] OR "qigong"[Title/Abstract] OR "T ai Chi"[Title/Abstract] OR "Tai Chi"[Title/Abstract] OR "Tai ji"[Title/Abstract] OR "Taiji"[Title/Abstract] OR "Taijiquan"[Title/Abstract] OR "traditional Chinese exercise"[Title/Abstract] OR "Wu Qin Xi"[Title/Abstract] OR "Wuqinxi"[Title/Abstract] OR "Yi Jin Jing"[Title/Abstract] OR "Yijinjing"[Title/Abstract] | 4,809 |
| 5 | (("Tai Ji"[Mesh]) OR ("Qigong"[Mesh])) OR ("Ba Duan Jin"[Title/Abstract] OR "Baduanjin"[Title/Abstract] OR "Ch i Kung"[Title/Abstract] OR "chi kung"[Title/Abstract] OR "chigung"[Title/Abstract] OR "Chinese traditional exercise"[Title/Abstract] OR "Dao Yin"[Title/Abstract] OR "Daoyin"[Title/Abstract] OR "Five Animal Frolics"[Title/Abstract] OR "health qigong"[Title/Abstract] OR "Liu Zi Jue"[Title/Abstract] OR "Liuzijue"[Title/Abstract] OR "mind body exercise"[Title/Abstract] OR "qi gong"[Title/Abstract] OR "qigong"[Title/Abstract] OR "T ai Chi"[Title/Abstract] OR "Tai Chi"[Title/Abstract] OR "Tai ji"[Title/Abstract] OR "Taiji"[Title/Abstract] OR "Taijiquan"[Title/Abstract] OR "traditional Chinese exercise"[Title/Abstract] OR "Wu Qin Xi"[Title/Abstract] OR "Wuqinxi"[Title/Abstract] OR "Yi Jin Jing"[Title/Abstract] OR "Yijinjing"[Title/Abstract]) | 4,903 |
| 6 | "random*"[Title/Abstract] OR "RCT"[Title/Abstract] OR "randomized controlled trial"[Title/Abstract] OR "control*"[Title/Abstract] OR "placebo"[Title/Abstract] | 6,241,888 |
| 7 | (("undergraduate"[Title/Abstract] OR "postgraduate"[Title/Abstract] OR "freshman"[Title/Abstract] OR "student*"[Title/Abstract]) AND ("Ba Duan Jin"[Title/Abstract] OR "Baduanjin"[Title/Abstract] OR "Ch i Kung"[Title/Abstract] OR "chi kung"[Title/Abstract] OR "chigung"[Title/Abstract] OR "Chinese traditional exercise"[Title/Abstract] OR "Dao Yin"[Title/Abstract] OR "Daoyin"[Title/Abstract] OR "Five Animal Frolics"[Title/Abstract] OR "health qigong"[Title/Abstract] OR "Liu Zi Jue"[Title/Abstract] OR "Liuzijue"[Title/Abstract] OR "mind body exercise"[Title/Abstract] OR "qi gong"[Title/Abstract] OR "qigong"[Title/Abstract] OR "T ai Chi"[Title/Abstract] OR "Tai Chi"[Title/Abstract] OR "Tai ji"[Title/Abstract] OR "Taiji"[Title/Abstract] OR "Taijiquan"[Title/Abstract] OR "traditional Chinese exercise"[Title/Abstract] OR "Wu Qin Xi"[Title/Abstract] OR "Wuqinxi"[Title/Abstract] OR "Yi Jin Jing"[Title/Abstract] OR "Yijinjing"[Title/Abstract])) AND ("random*"[Title/Abstract] OR "RCT"[Title/Abstract] OR "randomized controlled trial"[Title/Abstract] OR "control*"[Title/Abstract] OR "placebo"[Title/Abstract]) | 102 |

(2) Search History of web

| Search number | Query | Results |
| --- | --- | --- |
| 1 | ((TS=((undergraduate) OR (postgraduate) OR (freshman) OR (student*))) AND TS=((Ba Duan Jin) OR (Baduanjin) OR (Ch i Kung) OR (chi kung) OR (chigung) OR (Chinese traditional exercise) OR (Dao Yin) OR (Daoyin) OR (Five Animal Frolics) OR (health qigong) OR (Liu Zi Jue) OR (Liuzijue) OR (mind body exercise) OR (qi gong) OR (qigong) OR (T ai Chi) OR (Tai Chi) OR (Tai ji) OR (Taiji) OR (Taijiquan) OR (traditional Chinese exercise) OR (Wu Qin Xi) OR (Wuqinxi) OR (Yi Jin Jing) OR (Yijinjing))) AND ALL=((random*) OR (RCT) OR (randomized controlled trial) OR (control*) OR (placebo)) | 262 |

(3) Search History of Cochrane

| Search number | Query | Results |
| --- | --- | --- |
| 1 | MeSH descriptor: [Tai Ji] explode all trees | 638 |
| 2 | MeSH descriptor: [Qigong] explode all trees | 196 |
| 3 | (‘undergraduate’ OR ‘postgraduate’ OR ‘freshman’ OR ‘student*’):ti,ab,kw | 58106 |
| 4 | (‘Ba Duan Jin’ OR ‘Baduanjin’ OR ‘Ch i Kung’ OR ‘chi kung’ OR ‘chigung’ OR ‘Chinese traditional exercise’ OR ‘Dao Yin’ OR ‘Daoyin’ OR ‘Five Animal Frolics’ OR ‘health qigong’ OR ‘Liu Zi Jue’ OR ‘Liuzijue’ OR ‘mind body exercise’ OR ‘qi gong’ OR ‘qigong’ OR ‘T ai Chi’ OR ‘Tai Chi’ OR ‘Tai ji’ OR ‘Taiji’ OR ‘Taijiquan’ OR ‘traditional Chinese exercise’ OR ‘Wu Qin Xi’ OR ‘Wuqinxi’ OR ‘Yi Jin Jing’ OR ‘Yijinjing’):ti,ab,kw | 5009 |
| 5 | (‘random*’ OR ‘RCT’ OR ‘randomized controlled trial’ OR ‘control*’ OR ‘placebo’):ti,ab,kw | 1817777 |
| 6 | #1 OR #2 | 807 |
| 7 | #4 OR #6 | 5009 |
| 8 | #3 AND #7 | 269 |
| 9 | #5 AND #8 245 | 245 |

(4) Search History of Embase

| Search number | Query | Results |
| --- | --- | --- |
| 1 | 'undergraduate':ti,ab,kw OR 'postgraduate':ti,ab,kw OR 'freshman':ti,ab,kw OR  'student*':ti,ab,kw | 611,963 |
| 2 | 'ba duan jin':ti,ab,kw OR 'baduanjin':ti,ab,kw OR  'ch i kung':ti,ab,kw OR 'chi kung':ti,ab,kw OR  'chigung':ti,ab,kw OR 'chinese traditional  exercise':ti,ab,kw OR 'dao yin':ti,ab,kw OR  'daoyin':ti,ab,kw OR 'five animal  frolics':ti,ab,kw OR 'health qigong':ti,ab,kw OR  'liu zi jue':ti,ab,kw OR 'liuzijue':ti,ab,kw OR  'mind body exercise':ti,ab,kw OR 'qi  gong':ti,ab,kw OR 'qigong':ti,ab,kw OR 't ai  chi':ti,ab,kw OR 'tai chi':ti,ab,kw OR 'tai  ji':ti,ab,kw OR 'taiji':ti,ab,kw OR  'taijiquan':ti,ab,kw OR 'traditional chinese  exercise':ti,ab,kw OR 'wu qin xi':ti,ab,kw OR  'wuqinxi':ti,ab,kw OR 'yi jin jing':ti,ab,kw OR  'yijinjing':ti,ab,kw | 7,031 |
| 3 | 'random*':ti,ab,kw OR 'rct':ti,ab,kw OR  'randomized controlled trial':ti,ab,kw OR  'control*':ti,ab,kw OR 'placebo':ti,ab,kw | 8,551,052 |
| 4 | #1 AND #2 AND #3 | 168 |

(5) Search History of CBM

| Search number | Query | Results |
| --- | --- | --- |
| 1 | ( "大学生"[常用字段:智能] OR "高校学生"[常用字段:智能] OR "大学新生"[常用字段:智能] OR "本科生"[常用字段:智能] OR "研究生"[常用字段:智能] OR "高等院校学生"[常用字段:智能]) | 488401 |
| 2 | ( "太极"[常用字段:智能] OR "八段锦"[常用字段:智能] OR "易筋经"[常用字段:智能] OR "五禽戏"[常用字段:智能] OR "六字诀"[常用字段:智能] OR "气功"[常用字段:智能] OR "导引养生功"[常用字段:智能] OR "传统健身功法"[常用字段:智能] OR "传统功法"[常用字段:智能] OR "传统身心运动"[常用字段:智能] OR "传统运动疗法"[常用字段:智能]) | 39945 |
| 3 | "随机"[常用字段:智能] | 2442943 |
| 4 | #1 AND #2 AND #3 | 206 |

(6) Search History of CNKI

| Search number | Query | Results |
| --- | --- | --- |
| 1 | (篇关摘:大学生+高校学生+大学新生+本科生+研究生+高等院校学生(模糊))AND(篇关摘:太极+八段锦+易筋经+五禽戏+六字诀+气功+导引养生功+传统健身功法+传统功法+传统身心运动+传统运动疗法(模糊))AND(全文:随机(精确)) | 1085 |

(7) Search History of 维普

| Search number | Query | Results |
| --- | --- | --- |
| 1 | [(((((((任意字段=大学生 OR 任意字段=高校学生) OR 任意字段=大学新生) OR 任意字段=本科生) OR 任意字段=研究生) OR 任意字段=高等院校学生) AND ((((((((((任意字段=太极 OR 任意字段=八段锦) OR 任意字段=易筋经) OR 任意字段=五禽戏) OR 任意字段=六字诀) OR 任意字段=气功) OR 任意字段=导引养生功) OR 任意字段=传统健身功法) OR 任意字段=传统功法) OR 任意字段=传统身心运动) OR 任意字段=传统运动疗法)) AND 任意字段=随机)](https://qikan.cqvip.com/Qikan/search/index?LngMySearHistoryIdGuid=60836863-9ba5-4630-98c8-a6b1bb976d05&from=Qikan_Article_History) | 246 |

(8) Search History of 万方

| Search number | Query | Results |
| --- | --- | --- |
| 1 | 1981-[全部:(大学生 OR 高校学生 OR 大学新生 OR 本科生 OR 研究生 OR 高等院校学生) and 全部:(太极 OR 八段锦 OR 易筋经 OR 五禽戏 OR 六字诀 OR 气功 OR 导引养生功 OR 传统健身功法 OR 传统功法 OR 传统身心运动 OR 传统运动疗法) and 全部:(随机)](https://s.wanfangdata.com.cn/advanced-search/paper?q=%E5%85%A8%E9%83%A8%3A(%E5%A4%A7%E5%AD%A6%E7%94%9F%20OR%20%E9%AB%98%E6%A0%A1%E5%AD%A6%E7%94%9F%20OR%20%E5%A4%A7%E5%AD%A6%E6%96%B0%E7%94%9F%20OR%20%E6%9C%AC%E7%A7%91%E7%94%9F%20OR%20%E7%A0%94%E7%A9%B6%E7%94%9F%20OR%20%E9%AB%98%E7%AD%89%E9%99%A2%E6%A0%A1%E5%AD%A6%E7%94%9F)%20and%20%E5%85%A8%E9%83%A8%3A(%E5%A4%AA%E6%9E%81%20OR%20%E5%85%AB%E6%AE%B5%E9%94%A6%20OR%20%E6%98%93%E7%AD%8B%E7%BB%8F%20OR%20%E4%BA%94%E7%A6%BD%E6%88%8F%20OR%20%E5%85%AD%E5%AD%97%E8%AF%80%20OR%20%E6%B0%94%E5%8A%9F%20OR%20%E5%AF%BC%E5%BC%95%E5%85%BB%E7%94%9F%E5%8A%9F%20OR%20%E4%BC%A0%E7%BB%9F%E5%81%A5%E8%BA%AB%E5%8A%9F%E6%B3%95%20OR%20%E4%BC%A0%E7%BB%9F%E5%8A%9F%E6%B3%95%20OR%20%E4%BC%A0%E7%BB%9F%E8%BA%AB%E5%BF%83%E8%BF%90%E5%8A%A8%20OR%20%E4%BC%A0%E7%BB%9F%E8%BF%90%E5%8A%A8%E7%96%97%E6%B3%95)%20and%20%E5%85%A8%E9%83%A8%3A(%E9%9A%8F%E6%9C%BA)&searchtype=expert&type=%5b%22periodical%22,%22thesis%22%5d&chineseEnglishExpand=true) | 1981 |

**File S3** Heterogeneity analysis

(1) BMI

(2) vital capacity

(3) anxiety

(4) step test index

(5) body fat rate

(6) depression

(7) VO2 max

**Table S1** Consistency test results.

| Outcome | DIC | | ΔDIC |  |
| --- | --- | --- | --- | --- |
|  | Consistency | Inconsistency |  |  |
| BMI | 115.1 | 116.21 | 1.11 | 1.11 |
| BFR | 40.8 | 43.5 | 2.7 | 2.7 |
| VC | 108.4 | 111.8 | 3.4 | 3.4 |
| Vomax | 27.3 | 27.8 | 0.5 | 0.5 |
| Step Exponent | 53.2 | 53.2 | 0 | 0 |
| Anxiety | 110.2 | 113.74 | 3.54 | 3.54 |
| Depression | 170.38 | 170.76 | 0.38 | 0.38 |

**Table S2** SUCRA results.

| Treatment | Depression | Anxiety | VC | VO₂max | Step test index | BFR | BMI |
| --- | --- | --- | --- | --- | --- | --- | --- |
| BC | 28.64% | 35.77% | 22.07% | 12.29% | 6.36% | 5.66% | 17.22% |
| BDJ | 69.23% | 56.78% | 65.30% | 0.00% | 27.91% | 53.39% | 55.96% |
| DYYS | 37.72% | 48.27% | 0.00% | 0.00% | 0.00% | 0.00% | 0.00% |
| LZJ | 0.00% | 0.00% | 0.00% | 0.00% | 26.34% | 0.00% | 81.27% |
| LZJPI | 37.45% | 0.00% | 65.82% | 0.00% | 0.00% | 0.00% | 0.00% |
| MWDY | 0.00% | 0.00% | 0.00% | 82.07% | 0.00% | 0.00% | 40.48% |
| OTE | 0.00% | 48.12% | 84.53% | 23.11% | 82.52% | 0.00% | 48.62% |
| PI | 9.61% | 44.00% | 10.59% | 0.00% | 73.88% | 0.00% | 65.44% |
| RE | 53.08% | 25.50% | 42.81% | 56.12% | 63.24% | 54.60% | 45.74% |
| TC | 95.40% | 88.82% | 49.58% | 43.53% | 40.01% | 86.35% | 48.02% |
| WQX | 45.70% | 42.13% | 67.72% | 0.00% | 0.00% | 0.00% | 72.38% |
| XYQ | 37.75% | 51.84% | 29.70% | 82.89% | 78.81% | 0.00% | 35.04% |
| YJJ | 85.40% | 59.78% | 61.87% | 0.00% | 50.93% | 0.00% | 39.82% |
